# A systematic review and meta-analysis of host genetic factors associated with influenza severity

**DOI:** 10.1186/s12864-021-08240-7

**Published:** 2021-12-20

**Authors:** Nina Van Goethem, Célestin Danwang, Nathalie Bossuyt, Herman Van Oyen, Nancy H. C. Roosens, Annie Robert

**Affiliations:** 1 Scientific Directorate of Epidemiology and Public Health, Sciensano, J. Wytsmanstraat 14, 1050 Brussels, Belgium; 2 Department of Epidemiology and Biostatistics, Institut de Recherche Expérimentale et Clinique, Faculty of Public Health, Université Catholique de Louvain, Clos Chapelle-aux-champs 30, 1200 Brussels, Belgium; 3 Department of Public Health and Primary Care, Ghent University, De Pintelaan 185, 9000 Ghent, Belgium; 4 Transversal Activities in Applied Genomics, Sciensano, J. Wytsmanstraat 14, 1050 Brussels, Belgium

**Keywords:** Polymorphism, Influenza severity, Association, meta-analysis

## Abstract

**Background:**

The severity of influenza disease can range from mild symptoms to severe respiratory failure and can partly be explained by host genetic factors that predisposes the host to severe influenza. Here, we aimed to summarize the current state of evidence that host genetic variants play a role in the susceptibility to severe influenza infection by conducting a systematic review and performing a meta-analysis for all markers with at least three or more data entries.

**Results:**

A total of 34 primary human genetic association studies were identified that investigated a total of 20 different genes. The only significant pooled ORs were retrieved for the rs12252 polymorphism: an overall OR of 1.52 (95% CI [1.06–2.17]) for the rs12252-C allele compared to the rs12252-T allele. A stratified analysis by ethnicity revealed opposite effects in different populations.

**Conclusion:**

With exception for the rs12252 polymorphism, we could not identify specific genetic polymorphisms to be associated with severe influenza infection in a pooled meta-analysis. This advocates for the use of large, hypothesis-free, genome-wide association studies that account for the polygenic nature and the interactions with other host, pathogen and environmental factors.

**Supplementary Information:**

The online version contains supplementary material available at 10.1186/s12864-021-08240-7.

## Introduction

The severity of disease resulting from infection with influenza viruses can range from mild symptoms to severe respiratory failure. This variability is due to multiple host- and pathogen-related factors and the interplay between them. Clinical risk factors for severe influenza have been well documented [1–5]. Patient characteristics, such as age and the presence of comorbidities, are easily available for clinicians to estimate the risk of a patient to develop severe complications. Beside these, healthcare organization [6] and access to (intensive) care can significantly impact the outcome of severe acute respiratory infections, and partly explain differences in mortality rates across countries [7]. Disease severity also varies from influenza season to season [8], depending on the intrinsic pathogenicity of the type and subtype of circulating viruses. For example, subtype A(H3N2) usually causes more deaths than A(H1N1) [9, 10] or influenza B infections [10, 11], and it is often more severe in the elderly [12, 13]. We have recently demonstrated that adding viral genomic information to a predictive model on top of traditional clinical data, such as age and comorbidities, provides a more complete view on the predictors of severity of influenza infection, and results in an increased predictive accuracy [14]. However, a large part of the variation in disease severity observed between patients from the same country and within the same season remains unexplained. As in many other infectious diseases [15–17], host susceptibility to severe influenza disease is partly determined by specific host genes [18–20] that are involved in viral replication [21], interferon response [15], or virus-induced systemic inflammation. Indeed, numerous genes potentially participate in diverse mechanisms against the viral response [22]. The World Health Organization (WHO) prioritizes the identification of host genetic factors that predispose the host to severe influenza [23]. This leads to a better understanding of the biological mechanisms behind the development of severe disease, which may reveal new therapeutic approaches and may enable effective targets for vaccine therapies. Thereby, future clinically useful predictive models should include host genetic determinants together with traditional information on host characteristics and viral genomic data. However, the identification of a polygenic predisposition requires a large sample size, both because of the small expected effect attributable to individual variants and because of the additive nature of these genetic effects [24].

Candidate genetic markers typically only demonstrate small effects in genetic association tests [25, 26]. Consequently, larger sample sizes or a meta-analysis that combines results from previous studies on the same exposure-outcome relationship, are necessary to detect those small or moderate genetic effects of polymorphisms [25]. We aimed at providing a systematic review of all studies addressing a relationship between host gene variants and susceptibility to severe influenza infection. Additionally, we aimed at finding a common effect across studies focusing on the same genetic variant.

## Results

### Search results

**Fig. 1:**
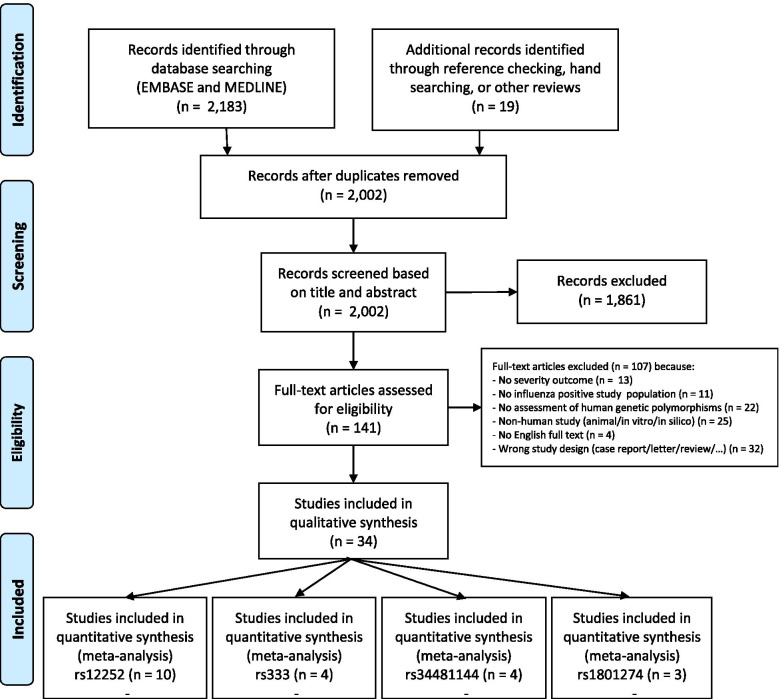
Preferred Reporting Items for Systematic Reviews and Meta-Analyses (PRISMA) flow diagram presenting the selection of studies included in the systematic review and meta-analysis of host genetic factors associated with influenza disease severity

The study selection process is summarized as a PRISMA flow diagram in Fig. 1. A total of 1983 de-duplicated records were retrieved from the initial bibliographic database search. In addition, 19 records were identified through reference checking, hand searching and other reviews. A total of 34 manuscripts were included in the systematic review after the first and second screening round. From these, a total of 74 data points, of which 71 with available genotype counts, were extracted. A total of 15 studies had multiple data points as they investigated multiple genotypes and/or there was a possibility to perform multiple post-hoc stratifications of cases and controls according to severity. There was a total of four genetic polymorphisms that had at least three data entries and for which a meta-analysis was conducted.

### Study characteristics

Additional file 1 presents the data extraction for all data points (*n* = 74), including the study characteristics, CSI score, genotype frequencies, HWE in controls, and re-calculated ORs. There were a total of 20 different genes assessed by the included studies (*n* = 34), with most frequent results for interferon-induced transmembrane protein 3 (*IFITM3*) (n studies = 11; 32%). Other genes investigated were C-C motif chemokine receptor 5 (*CCR5*, *n* = 4), Fc fragment of IgG receptor IIa (*FCGR2A*, *n* = 3), tumor necrosis factor (*TNF*, *n* = 1), toll-like receptor (*TLR2/TLR3/TLR4*, *n* = 2), complement decay-accelerating factor (*CD55*, *n* = 2), surfactant protein (*SFTPA1/SFTPA2/SFTPB*, *n* = 2), ST3 beta-galactoside alpha-2,3-sialyltransferase 1 (*ST3GAL1*, *n* = 1), angiotensin I converting enzyme (*ACE*, *n* = 1), complement C1q binding protein (*C1QBP*, *n* = 1), C-X-C motif chemokine ligand 14 (*CXCL14*, *n* = 1), glycine decarboxylase (*GLDC*, *n* = 1), mannose binding lectin 2 (*MBL2*, *n* = 1), natural killer gene (*NKG2C*, *n* = 1), transmembrane serine protease 2 (*TMPRSS2*, *n* = 1), and phosphoinositide-3 kinase (PI3K, *n* = 1). A total of 34 distinct markers were recorded in the database, but only four met the inclusion criteria of at least three data points available per marker and were used in the subsequent meta-analysis. The majority of included studies (76%) was performed in influenza A(H1N1)pdm09 positive patients. Among investigated data points, a total of 19 (26%) investigated severity indicators (e.g., ICU admission, invasive ventilation or mortality) among hospitalized patients, 11 (15%) focused specifically on mortality among hospitalized patients, 10 (14%) on mortality among hospitalized and ambulant patients combined, 17 (23%) on severity indicators among hospitalized and ambulant patients combined, and 17 (23%) assessed the risk for hospitalization. From the total of 74 data points, 40 (54%) were characterized by a post-hoc stratification of cases and controls. Out of the 34 data points with all three CSI score domains scored, only 5 (15%) had a credible CSI score (i.e., having only A or B grades).

### Meta-analyses

**Fig. 2:**
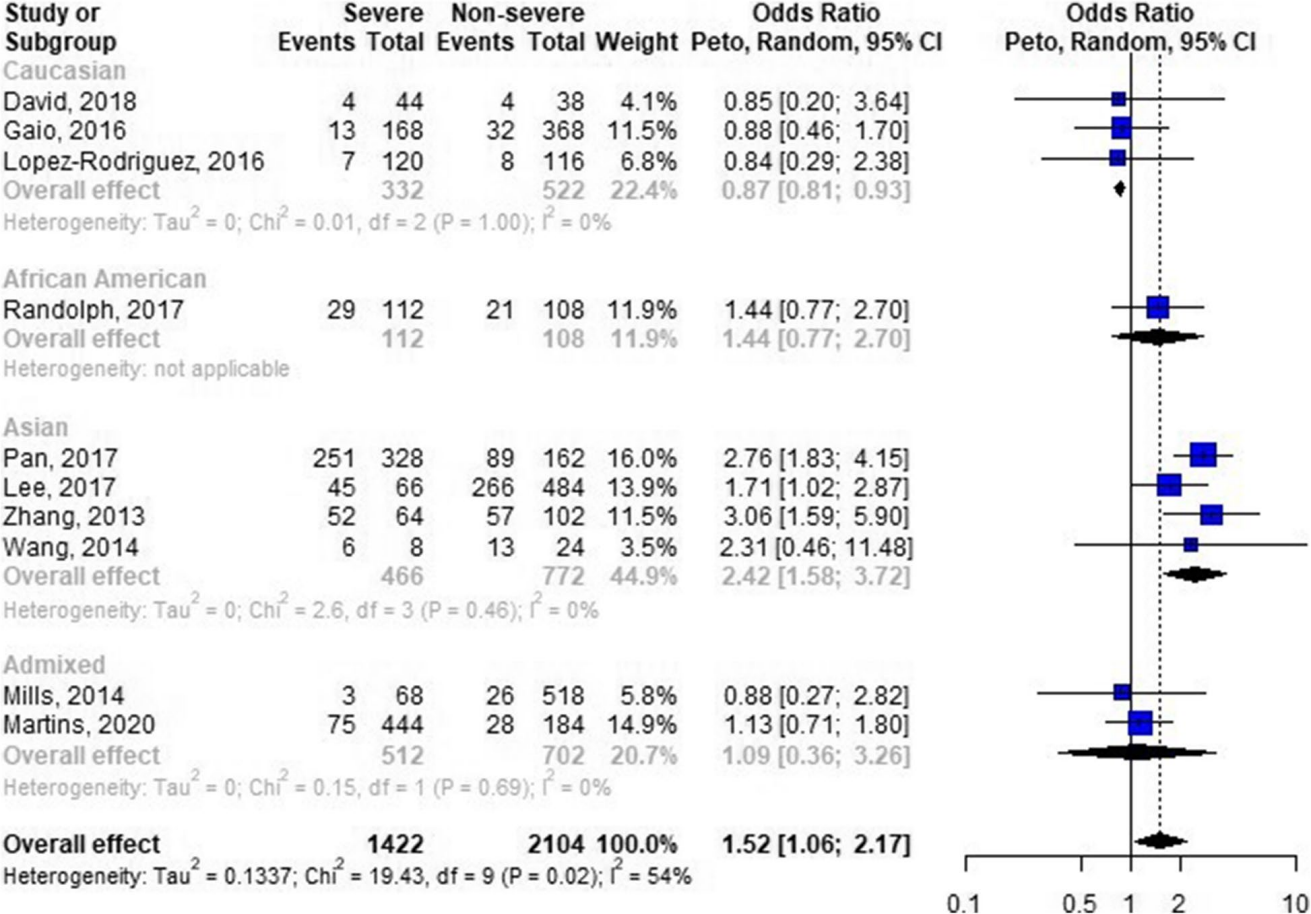
Forest plot of the odds ratios of the *IFITM3* rs12252 polymorphism and influenza disease severity in the allelic model, stratified per ethnicity. The random effect model was used to compute a common effect size

We performed a meta-analysis for four different markers, rs12252 and rs34481144 in the *IFITM3* gene, rs333 in the *CCR5* gene, and rs1801274 in the *FCGR2A* gene, in all four genetic models. Table 1 shows the noteworthy results for each of the markers. The only significant pooled odds ratios were retrieved for the rs12252 polymorphism from the *IFITM3* gene. In the homozygous model, the odds for severe influenza disease was multiplied by 2.68 (95% CI [1.22–5.88]) when having the C/C genotype as compared to the T/T genotype. Similar results were obtained in the allelic model, with an overall OR of 1.52 (95% CI [1.06–2.17]) for the rs12252-C allele compared to the rs12252-T allele, albeit with more heterogeneity across studies (I^2^ = 54%). Indeed, a stratified analysis by ethnicity revealed opposite effects in different populations: there was a 2.42-fold (95% CI [1.58–3.72]) increase in odds for severe influenza disease when having the rs12252-C allele within an Asian population, whereas the odds for severe influenza diseases when having the rs12252-C allele was decreased by 13% within a Caucasian population (OR = 0.87, 95% CI [0.81–0.93]) (Fig. 2). The pooled ORs were non-significant for the rs333, rs1801274, and rs34481144 polymorphisms in all genetic models and subgroup analyses. The leave-one-out analyses did not detect influential data points, nor did it change the conclusions. We also did not detect strong indications of publication bias in any of the meta-analyses. All meta-analyses, and the corresponding subgroup analyses, sensitivity analyses, and funnel plots, are available in Additional file 2.

**Table 1:** Noteworthy results of meta-analyses assessing the association between host genetic factors and influenza disease severity. The entire set of results with meta-analyses performed using four different genetic models and stratified per ethnicity, study population or severity definition are available in Additional file 2

Gene	rs code	Genetic model	Heterozygote	Risk allele	Studies (n)	Cases (alleles) (n)	Controls (alleles) (n)	OR^**a**^ [95% CI]	I^**2**^	Venice score
IFITM3	rs12252	Allelic	CT	C	10	1422	2104	1.52 [1.06; 2.17]	54%	BCC
IFITM3	rs12252	Homozygous	CT	C	10	1422	2104	2.68 [1.22; 5.88]	29%	BBC
CCR5	rs333	Dominant	wt/Δ32	Δ32	4	934	990	1.20 [0.10; 13.83]	68%	BCC
FCGR2A	rs1801274	Allelic	AG	G	3	680	558	1.03 [0.70; 1.53]	0%	BAC
IFITM3	rs34481144	Allelic	AG	A	4	540	872	1.47 [0.24; 9.00]	83%	BCC

*OR* Odds ratio, *IFITM3* Interferon-induced transmembrane protein 3, *CCR5* C-C motif chemokine receptor 5, *FCGR2A* Fc fragment of IgG receptor IIa

^a^Pooled random effect odds ratio calculated using Peto’s method

## Discussion

This systematic review and meta-analysis aimed to summarize the published evidence on the role of human genetic factors in influenza disease severity and subsequently sought a common effect across studies focusing on the same genetic variant. Several host genetic polymorphisms related to susceptibility to severe influenza disease have been studied and reported with varying levels of evidence. The included primary human genetic association studies, conducted within influenza positive patient cohorts, investigated whether patients with increased susceptibility to severe influenza disease had impaired intracellular control of viral replication (e.g. *IFITM3* [27–37], *TMPRSS2* [38] variants), defective interferon responses (e.g. *GLDC* [39] variants), polymorphisms in genes implicated in the cytokine response (e.g. *TNF* [40], *CCR5* [41–44] variants), dysfunction of proteins related to the complement cascade (e.g. *CD55* [45, 46], *C1QBP* [45] variants), variability at the collectin genes (e.g. *MBL2*, *SFTPA1*, *SFTPA2* variants [47–49]), or polymorphisms affecting the affinity of the immunoglobulin Fc receptor (*FCGR2A* [45, 50, 51]) or pattern-recognition receptors (e.g. *TLR3* [30, 52], *TLR2* [52], *TLR4* [52] variants). Although some of the primary studies found evidence for a significant association between the human genetic polymorphism and influenza disease severity, only few associations were replicated within multiple independent cohorts.

We did find a significant pooled association for the *IFITM3* SNP rs12252-C allele and severity of influenza infection, which is in line with previously published meta-analyses [53–56]. The protein product of *IFITM3* restricts viral entry by blocking the fusion of host and viral membranes [57] and acts as a restriction factor affecting several stages of the viral replication cycle [58]. However, the prevalence of the rs12252-C allele is heterogeneously distributed among populations. Population-level studies suggested that this allele is rare in European cohorts, but frequent in the Han Chinese cohorts hospitalized with severe H1N1pdm09 infection [27, 33, 34, 59]. The higher allele frequency of *IFITM3* rs12252-C within the Han Chinese population may greatly increase the power of analysis [60] and partly explain the significant association found within these primary studies [29, 30, 33, 35], as opposed to studies conducted within European or African-American patient cohorts where the minor allele frequency is much lower and resulted in non-significant associations within these primary studies [27, 28, 32, 37]. Strikingly, the meta-analysis stratified per ethnicity found a protective effect of the rs12252-C allele in the Caucasian population. However, these findings are not in line with other meta-analyses that consistently found a harmful effect of the rs12252-C allele across different ethnic populations, although they investigated susceptibility to influenza infection and used the general healthy population (or healthy population in the 1000 Genomes project) as the control group [53, 56]. Still, the observed opposite effects may be due to possible linkage disequilibrium with other variants at *IFITM3* or nearby genes of which the minor allele frequencies can also vary between populations [27, 29]. Therefore, the susceptibility to severe influenza infection should rather be investigated based on haplotypes of the *IFITM3* polymorphisms instead of single SNPs [59]. Further, the observed differences between populations in allele frequencies and the association with disease severity have implications for vaccination programs. Given the high frequency of the C/C genotype in the Chinese population, individuals in East Asia with a C/C genotype should be preferentially targeted for routine influenza vaccination [35].

Susceptibility to severe influenza in humans is likely to be polygenic and co-determined by viral characteristics, co-morbidities, and contextual factors [27]. In addition, the inconsistent evidence of the SNPs can be partially explained by the differences in the genetic background of the populations around the world [22]. As such, the lack of consistently replicated evidence within diverse populations pinpointing any specific genes in humans, advocates the use of large, hypothesis-free, genome-wide association studies [61]. Indeed, the potential role of individual polymorphisms might be diluted or masked by other gene-gene or gene-environment interactions [55]. Moreover, a single variant is often not informative for assessing disease risk given that genetic predisposition to most traits is spread and shared across a large number of variants [62]. Therefore, the effects of genetic variants should be investigated jointly by means of a genetic risk score [63]. The use of high-throughput sequencing technologies for human genetic association studies offers the ability to look at an increasing number of markers at once. Still, the inability to identify novel genetic risk factors for infectious diseases might be related to heterogeneity at the level of the infectious agent. Currently, the impact of host genetic variation is often studied separately from that of pathogen genomics. An integrative analysis, considering both host and viral genetic variation, will be highly informative [62].

Traditional journal formats often prevent the publication of summary data, such as genotype distributions, for each genetic marker that has been investigated in a genome-wide association study. Given these constraints, association studies investigating many genetic markers often only selectively report the most promising variants and existing results [30, 46–49, 64]. Next to selective reporting, reporting bias can also be the result of publication bias, which refers to suppression of evidence from an entire study based on its findings. Therefore, a literature-based systematic review can be prone to bias due to selective availability of published data [65].

Strengths of the current systematic review are the fact that the methodology, including eligibility criteria for study inclusion, search strategy, critical appraisal, and meta-analysis, was carefully planned with a detailed protocol prepared and registered in advance. In addition, the review was characterized by a comprehensive search without time limits, and which consulted both the MEDLINE and EMBASE bibliographic databases, as well as manual reference checking of included studies and existing reviews. The formulated inclusion and exclusion criteria were strictly applied and resulted in a homogenous collection of human genetic association studies. The review question focused on the severity of influenza infection, thereby excluding studies that only considered an influenza-negative (healthy) control group. As such, we required the case and control status to be determined by the severity of illness, which we consider as a more informative methodology for identifying genetic factors contributing to risk of severe illness as compared to studies comparing influenza-infected cases to healthy controls with unknown history of response to infection [31]. Previously published reviews were often restricted to a single polymorphism [53, 54, 56], assessed the susceptibility to influenza infection [66], or were non-systematic [19, 20].

The severity of influenza infection can be defined based on multiple criteria and within different study populations. Individual studies may measure “hard” outcomes, such as mortality, to assess disease severity, or they can be based on clinical decisions, such as admission to the hospital or ICU. The criteria to admit a patient to the hospital or ICU used in different health units might not be uniform and could differ according to hospital-specific policies, epidemic alerts, and the indications of health authorities [34]. Also, for some of the studies there was no follow-up to confirm the outcome of non-severely ill patients. These differences in end-point definitions may hamper the comparison of results between studies. However, this was taken into account by performing subgroup analyses based on the study population (e.g., hospitalized vs ambulant patients) or definition of severity (e.g., mortality vs other severity indicators), and by using a random effect model to calculate the common OR. In addition to varying outcome definitions and study populations, primary studies often use different inheritance models without a biological justification available [67]. Instead of assuming a particular inheritance model and subsequently obtaining a single effect estimate during the meta-analysis [68], we obtained pooled estimates using four different genetic models.

## Conclusion

The identification of host genetic factors, and their interaction with other host factors and viral characteristics, is of great importance for a better understanding of the biological predisposition of patients to severe complications. Here, we have identified several human genetic association studies leading to a significant effect of a genetic polymorphism and disease severity. However, with exception for the *IFITM3* rs12252 polymorphism, we could not pinpoint specific genetic polymorphisms to be associated with severe influenza infection in a pooled meta-analysis. This could be due to heterogeneity between studies, but also because of the polygenic nature of infectious disease pathogenicity advocating the use of large genome-wide association studies and subsequent analyses based on haplotypes. A better knowledge of the host-pathogen interactions underlying the pathophysiology of influenza could also help to better understand the mechanisms of other respiratory pathogens, such as SARS-CoV-2.

## Methods

The review was conducted in accordance with the guidelines from the HuGE review handbook for systematic review and meta-analysis of gene disease association studies [69] and was reported with respect to the Preferred Reporting Items for Systematic Reviews and Meta-Analyses (PRISMA) statement guidelines [70]. The protocol of the current review was registered on the PROSPERO International prospective register of systematic reviews (Trial Registration: CRD42020172583).

### Search strategy

The MEDLINE and EMBASE electronic bibliographic databases were searched to identify relevant studies published before 5 July 2021. Three domains were included in the search using the PubMed search engine for the MEDLINE database: “host genetics”, “influenza” and “severe infection”. Each domain has several search terms combining MeSH terms and free text search to ensure that manuscripts containing any variation of each of the search terms were identified. The predefined search strategy used in PubMed and its adapted form to fit with the EMBASE database are presented in Additional file 3. In addition, reference lists of included studies and other reviews were examined (i.e., backward snowballing).

### Eligibility criteria

Only primary studies enrolling individuals with a laboratory-confirmed influenza infection and allowing to compare severe cases with non-severe controls were considered. Severity had to be defined as: 1) severity indicators (e.g., intensive care unit (ICU) admission, invasive ventilation, or mortality) among hospitalized patients; 2) mortality among hospitalized patients; 3) mortality among hospitalized and ambulant patients; 4) severity indicators (e.g., ICU admission, invasive ventilation, or mortality) among hospitalized and ambulant patients; or 5) hospital admission among ambulant patients. Studies that investigated susceptibility to influenza infection itself, and thereby comparing influenza positive individuals to influenza negative individuals or healthy controls, were excluded. Further, this review only focused on studies conducted on human subjects within a real-life public health setting, so without taking into account animal, in vitro, or in silico studies. All genetic polymorphisms in the human genome, including bi-allelic single-nucleotide polymorphisms (SNPs) and bi-allelic insertion or deletion marker types, were considered as potential exposures. Studies based only on other marker types, such as microsatellites, short tandem repeats, aggregated haplotypes, or gene expression profiles were excluded. Finally, case reports, letters, comments, reviews, and editorials were excluded, as well as manuscript without an English full text available. A full list of exclusion and inclusion criteria can be found in Additional file 3. A first screening phase based on titles and abstracts was conducted [NVG] and out-of-topic studies were excluded. A second screening stage based on the full texts was conducted in duplicate by two independent reviewers [NVG, CD] using a standardized eligibility form. Any disagreement was solved by discussion. The studies that did match the eligibility criteria but that did not report the number of cases and controls for every genotype, as is often the case for genome-wide association studies (GWAS), were only included in the qualitative summary.

### Data extraction

The following information was retrieved on a preconceived data extraction form in duplicate by two independent reviewers [NVG, CD]: the name of the first author, the country where the study was conducted, the year of publication, the study design (cohort or case-control), the sampling period, the setting, the patient selection criteria, the characteristics of the study population in terms of ethnicity and age groups, the influenza subtype(s), a description of cases and controls based on the severity, the genotyping method, and the SNPs/deletions/insertions and corresponding human genes that were investigated, number of eligible cases and controls, and the frequency of the alleles and genotype distributions for both the cases and controls. Genotype counts were extracted as raw numbers or were calculated from the reported percentages and total sample sizes. If the distribution of alleles was not given, it was calculated by genotype distribution. Some studies provided genotype distributions for more than one marker and were consequently represented with more than one data point in our database. Similarly, when multiple cases or control groups could be defined based on the severity within one study, they were either pooled or retained as multiple data points from the same study with a clear description of cases and controls in the data extraction. All data points were classified according to the study population and/or underlying definition of severity.

### Quality assessment

The Confounding-Selection-Information (CSI) bias score developed by Patarčić et al [71] was used to evaluate the methodological quality of the included studies (Additional file 3). The elements of the score were developed on the basis of several existing assessment scores, including Venice criteria for assessing cumulative epidemiologic evidence in genetic associations [72], Newcastle-Ottawa case-control scale [73], and the Cochrane risk of bias tool [74]. The three domains, confounding, selection, and information, were scored in three grades of credibility: high – A; intermediate – B; or weak – C. This scheme was applied to every data point and provided estimates ranging from the best AAA to the worst CCC score. When there was a post-hoc stratification of the study population into cases and controls, we assigned 0 to selection bias risk. As an additional quality check, the Hardy-Weinberg Equilibrium (HWE) test was re-calculated for every control set included in the analysis. Data points that failed the HWE at the level *p* < 0.05 were downgraded to C in the CSI score.

Risk of bias across studies was assessed using the Venice criteria [72] for the noteworthy meta-analysis results. Study power was scored on the basis of sample size (graded as A when over 10,000, as B when 1000-10,000, and as C when less than 1000). Heterogeneity was based on the I^2^ statistic (graded as A when 0–25%, as B when 26–50%, and C when over 50%). The third score was fixed as C (weak credibility), given the high risk of bias in primary studies (as demonstrated in the Results section).

### Statistical analysis

The HWE was (re-)calculated for every control group of each included study using an exact test. A *p*-value < 0.05 was considered as indication of a significant departure from HWE. Odds ratios (ORs) and 95% confidence intervals (CI) were calculated anew from raw genotype and allele counts. Results from four different genetic models assuming different inheritance effects (allelic, heterozygous, homozygous, and dominant) were compared in order to provide broader insight into the underlying genetic architecture. In addition, a common OR and corresponding *p*-value based on the Armitage’s trend test was calculated [75, 76]. The calculated ORs correspond to taking the minor allele (with Minor Allele Frequency (MAF) < 0.5 in the control group) as the risk allele. The HWE and ORs were calculated for each data point using a web tool (http://ihg.gsf.de/cgi-bin/hw/hwa1.pl). A series of meta-analyses using four different genetic models (allelic, heterozygous, homozygous, and dominant) were performed for all markers where three or more data entries were available. For the purpose of the meta-analysis, the risk allele was uniformly defined for all data points based on literature, independently of the minor allele within individual studies. It was ensured that each data point in the meta-analysis represents an independent sample of data. When multiple cases or control groups were available in one study, the data point with the largest total sample size and/or focusing on severity (instead of only on mortality) was considered for the main analysis. The presence of heterogeneity was assessed using the Cochran’s Q-test and quantified with the *I*^*2*^ metric. A random effects model for meta-analysis was used to calculate the overall OR with their 95% CI assuming differences in designs, severity definitions and ethnicity of individual studies. Peto’s method was used to pool odds ratios [77]. Stratified analyses were performed, when appropriate, by ethnicity and the classification based on the underlying definition of severity and/or study population. Leave-one-out analyses were performed to detect influential data points. Funnel plots [78] were used to assess potential publication bias. The “meta” package within R software version 4.0.2 was used for the meta-analyses [79].

## Supplementary Information

**Additional file 1.** Data extraction of the included studies in the systematic review of host genetic factors and influenza disease severity.

**Additional file 2.** Results of the meta-analyses, leave-one-out analyses, and funnel plots for the association between the rs12252, rs333, rs1801274, and rs34481144 polymorphisms and influenza disease severity.

**Additional file 3.** Search strategy using the MEDLINE and EMBASE databases, eligibility criteria, and risk of bias score to assess the quality of included studies for the systematic review and meta-analysis of host genetic factors and influenza disease severity.

## Data Availability

The dataset(s) supporting the conclusions of this article is (are) included within the article (and its additional file(s)).

## Abbreviations

ACE: Angiotensin I converting enzyme
C1QBP: Complement C1q binding protein
CCR5: C-C motif chemokine receptor 5
CD55: Complement decay-accelerating factor
CI: Confidence interval
CSI: Confounding-Selection-Information
CXCL14: C-X-C motif chemokine ligand 14
FCGR2A: Fc fragment of IgG receptor IIa
GLDC: Glycine decarboxylase
GWAS: Genome-wide association studies
HWE: Hardy-Weinberg Equilibrium
ICU: Intensive care unit
IFITM3: Interferon-induced transmembrane protein 3
MAF: Minor allele frequency
MBL2: Mannose binding lectin 2
NGS: Next Generation Sequencing
NKG: Natural killer gene
OR: Odds ratio
PI3K: Phosphoinositide-3 kinase
PRISMA: Preferred Reporting Items for Systematic Reviews and Meta-Analyses
SARS-CoV-2: Severe acute respiratory syndrome coronavirus 2
SNP: Single Nucleotide Polymorphism
SFTP: surfactant protein
ST3GAL1: ST3 beta-galactoside alpha-2,3-sialyltransferase 1
TLR: Toll-like receptor
TMPRSS2: Transmembrane serine protease 2
TNF: Tumor necrosis factor
WGS: Whole-genome sequencing
WHO: World Health Organization
