# Supplementary material for: A systematic review and meta-analysis of host genetic factors associated with influenza severity
Source: BMC Genomics. 2021 Dec 20;22:912. doi: 10.1186/s12864-021-08240-7 (PMC8686082; doi:10.1186/s12864-021-08240-7)
Supplement: Supplementary file 2 — Additional file 2. Results of the meta-analyses, leave-one-out analyses, and funnel plots for the association between the rs12252, rs333, rs1801274, and rs34481144 polymorphisms and influenza disease severity. [file 12864_2021_8240_MOESM2_ESM.docx]

**Additional file 2**

**A meta-analysis of host genetic factors associated with influenza severity**

Nina Van Goethem^1,2, *^, Célestin Danwang^2^, Nathalie Bossuyt^1^, Herman Van Oyen^1,3^, Nancy H. C. Roosens^4,£^, Annie Robert^2,£^

^1^Scientific Directorate of Epidemiology and public health, Sciensano, J. Wytsmanstraat 14, 1050 Brussels, Belgium.

^2^Department of Epidemiology and Biostatistics, Institut de recherche expérimentale et clinique, Faculty of Public Health, Université catholique de Louvain, Clos Chapelle-aux-champs 30, 1200 Brussels, Belgium.

^3^Department of Public Health and Primary Care, Ghent University, De Pintelaan 185, 9000 Ghent, Belgium.

^4^Transversal activities in Applied Genomics, Sciensano, J. Wytsmanstraat 14, 1050 Brussels, Belgium.

^£^Shared last author

**Results meta-analysis IFITM3 – rs12252**

1. Study characteristics

| **Study** | **Ethnicity** | **Assessment of severity** | **Cases (n)** | **Controls (n)** | **rs12252 cases** | | | | **rs12252 controls** | | | | $\boldsymbol{P}_{\boldsymbol{HWE}}\boldsymbol{*}$ |
| --- | --- | --- | --- | --- | --- | --- | --- | --- | --- | --- | --- | --- | --- |
|  |  |  |  |  | **CC** | **CT** | **TT** | **MAF** | **CC** | **CT** | **TT** | **MAF** |  |
| David et al., 2018 | Caucasian | Severity indicators among H and non-H patients | 22 | 19 | 0 | 4 | 18 | 0.09 (C) | 1 | 2 | 16 | 0.11 (C) | 0.16 |
| Randolph et al., 2017 | African American | Severity indicators among H and non-H patients | 56 | 54 | 4 | 21 | 31 | 0.26 (C) | 1 | 19 | 34 | 0.19 (C) | 0.66 |
| Pan et al., 2017 | Asian | Hospital admission among H and non-H patients | 164 | 81 | 101 | 49 | 14 | 0.23 (T) | 22 | 45 | 14 | 0.45 (T) | 0.37 |
| Lee et al., 2017 | Asian | Mortality among H and non-H patients | 33 | 242 | 18 | 9 | 6 | 0.32 (T) | 79 | 108 | 55 | 0.45 (T) | 0.12 |
| Mills et al., 2014 | Admixed | Severity indicators among H and non-H patients | 34 | 259 | 0 | 3 | 31 | 0.04 (C) | 2 | 22 | 235 | 0.05 (C) | 0.12 |
| Zhang et al., 2013 | Asian | Severity indicators (e.g. ICU, ventilation, mortality) among H patients | 32 | 51 | 22 | 8 | 2 | 0.19 (T) | 13 | 31 | 7 | 0.44 (T) | 0.16 |
| Wang et al., 2014 | Asian | Mortality among H patients | 4 | 12 | 2 | 2 | 0 | 0.25 (T) | 4 | 5 | 3 | 0.46 (T) | 0.59 |
| Gaio et al., 2016 | Caucasian | Hospital admission among H and non-H patients | 84 | 184 | 2 | 9 | 73 | 0.08 (C) | 0 | 32 | 152 | 0.09 (C) | 0.37 |
| Lopez-Rodriguez et al., 2016 | Caucasian | Hospital admission among H and non-H patients | 60 | 58 | 0 | 7 | 53 | 0.06 (C) | 1 | 6 | 51 | 0.07 (C) | 0.23 |
| Lopez-Rodriguez et al., 2016 | Caucasian | Severity indicators (e.g. ICU, ventilation, mortality) among H patients | 34 | 26 | 0 | 5 | 29 | 0.07 (C) | 0 | 2 | 24 | 0.04 (C) | >0.99 |
| Martins et al., 2020 | Admixed | Severity indicators among H and non-H patients | 222 | 92 | 9 | 57 | 156 | 0.17 (C) | 3 | 22 | 67 | 0.15 (C) | 0.43 |
| Martins et al., 2020 | Admixed | Mortality among H and non-H patients | 82 | 232 | 0 | 27 | 55 | 0.16 (C) | 12 | 52 | 168 | 0.16 (C) | 0.01 |

*HWE calculated within controls.

H: hospitalized; HWE: Hardy-Weinberg Equilibrium; MAF: minor allele frequency.

1. Meta-analysis of the main outcome per study (main analysis)

| Allelic model (C *vs* T) |
| --- |
| 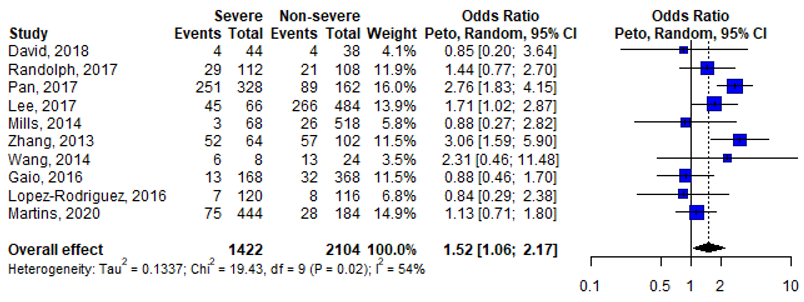 |
| Dominant model (CC/CT *vs* TT) |
| 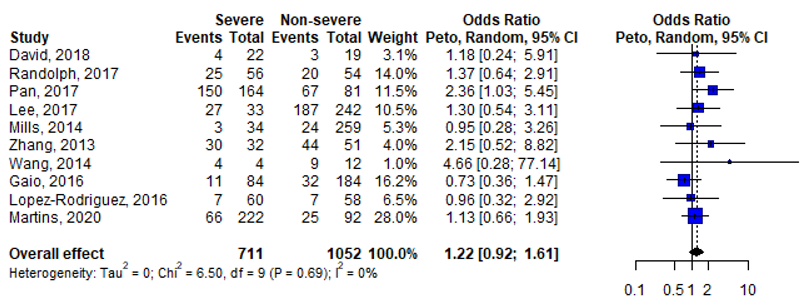 |
| Homozygous model (CC *vs* TT) |
| 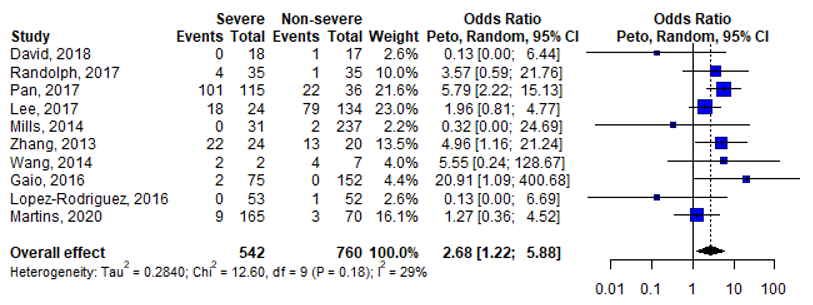 |

| Heterozygous model (CT *vs* TT) |
| --- |
| 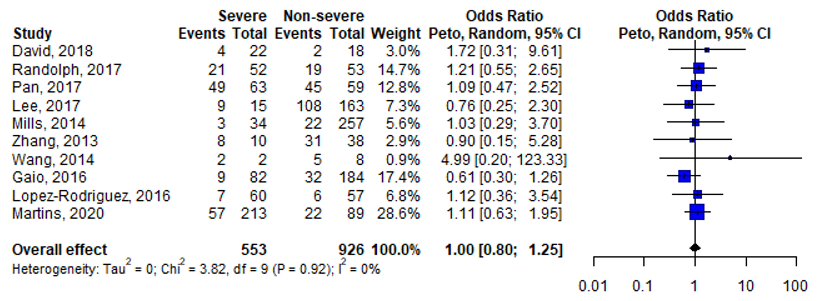 |

1. Meta-analysis of the main outcome per study (main analysis) stratified per ethnicity

| Allelic model (C *vs* T) |
| --- |
| 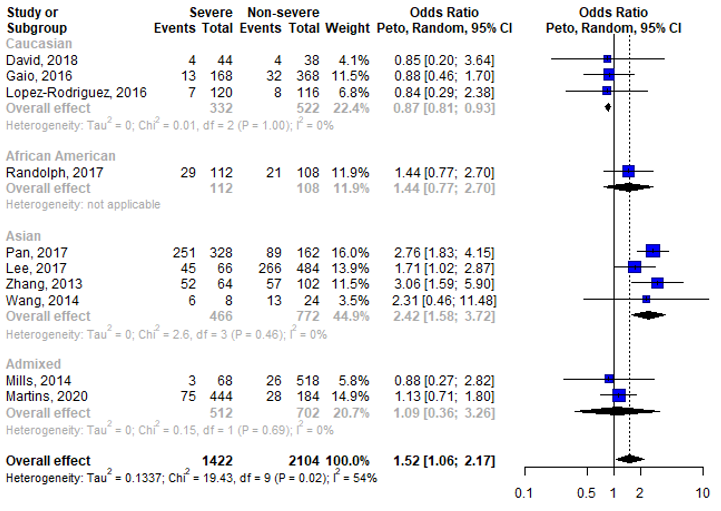 |

| Dominant model (CC/CT *vs* TT) |
| --- |
| 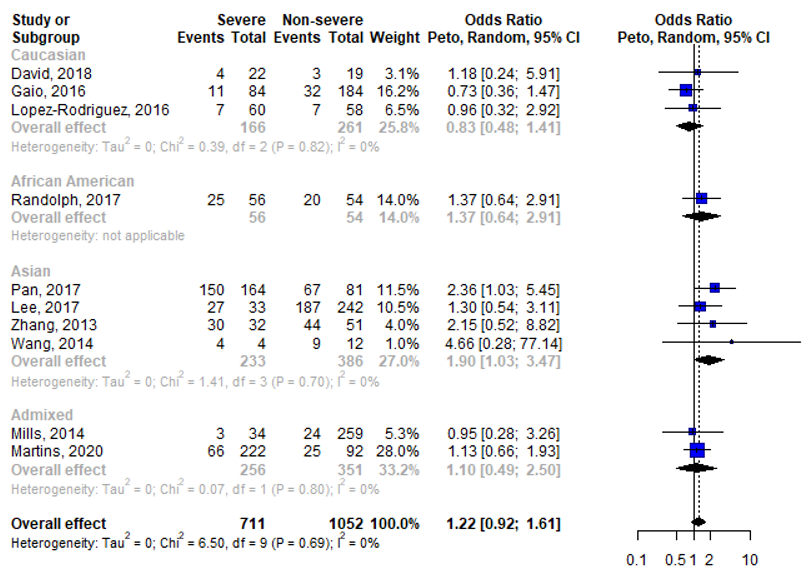 |
| Homozygous model (CC *vs* TT) |
| 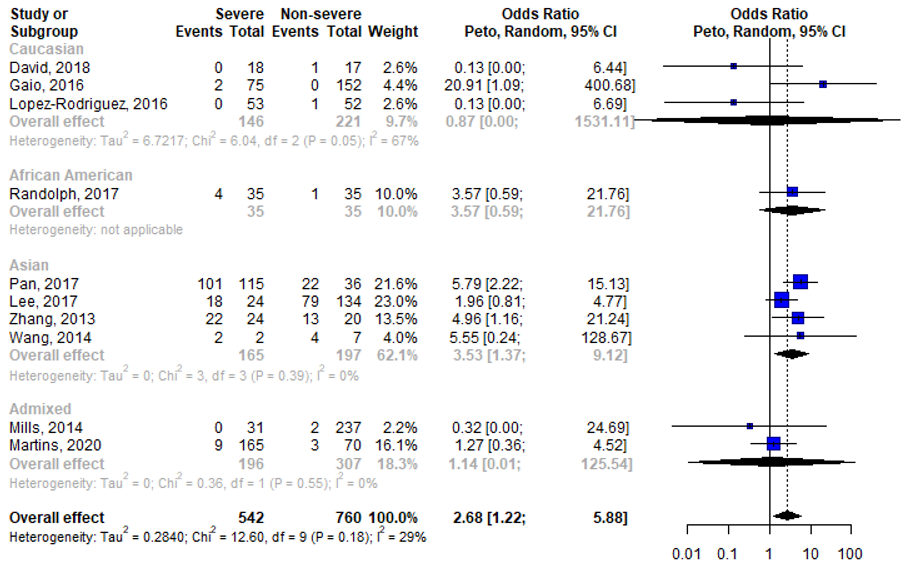 |

| Heterozygous model (CT *vs* TT) |
| --- |
| 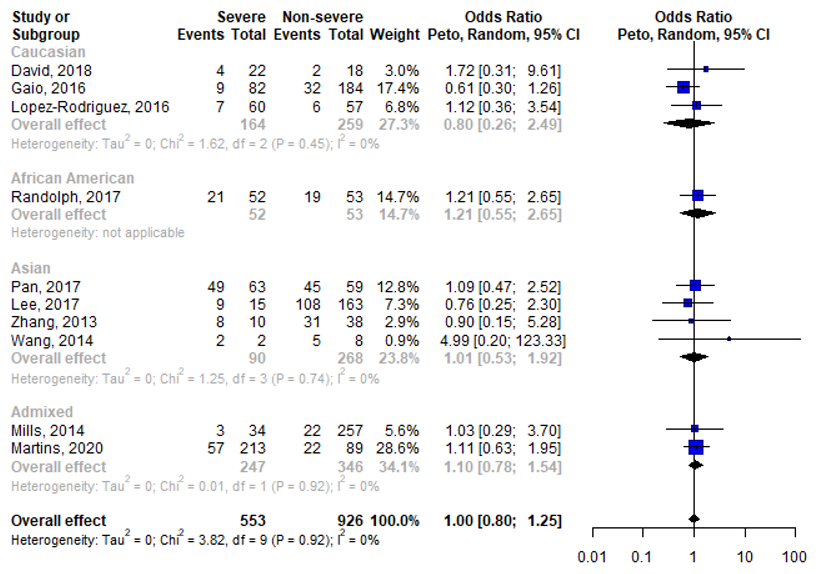 |

1. Meta-analysis stratified per study population and/or assessment of severity

| Allelic model (C *vs* T) |
| --- |
| 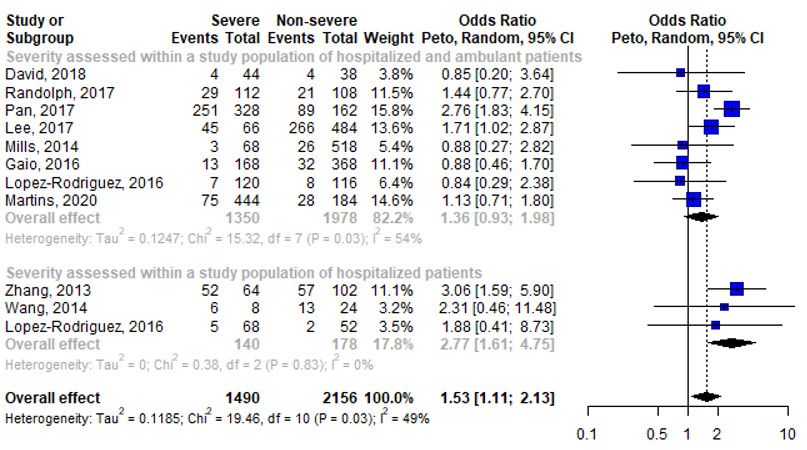 |

| Dominant model (CC/CT *vs* TT) |
| --- |
| 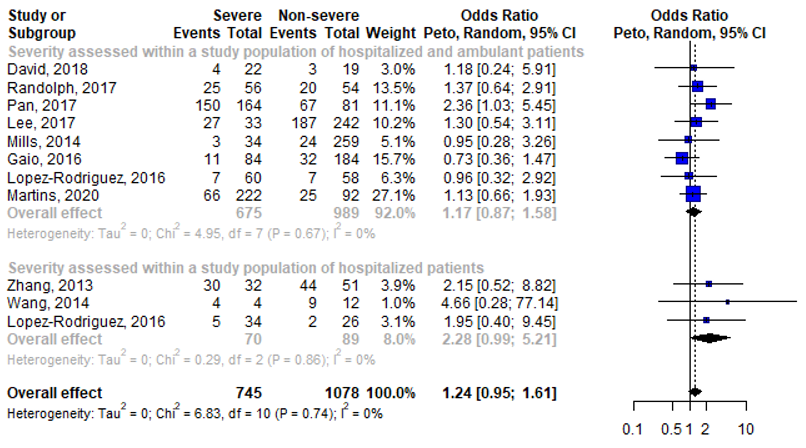 |
| Homozygous model (CC *vs* TT) |
| 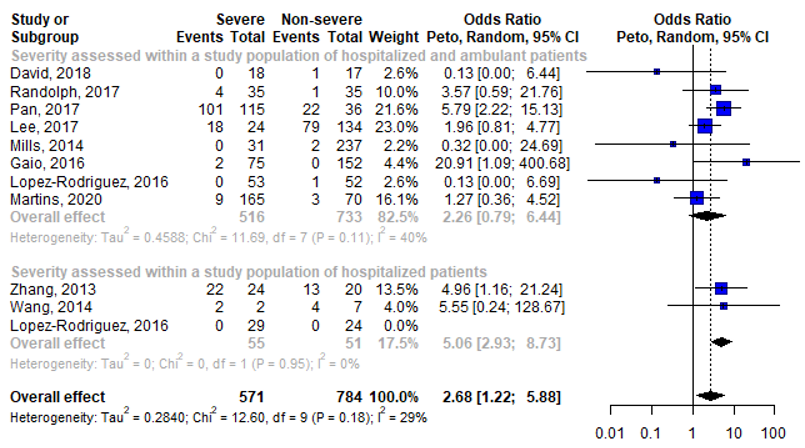 |

| Heterozygous model (CT *vs* TT) |
| --- |
| 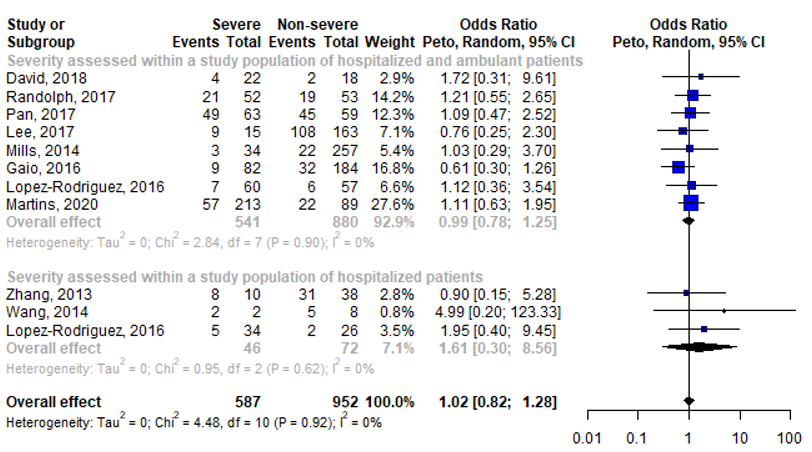 |

1. Leave-one-out analysis (sensitivity analysis)

| Allelic model (C *vs* T) |
| --- |
| 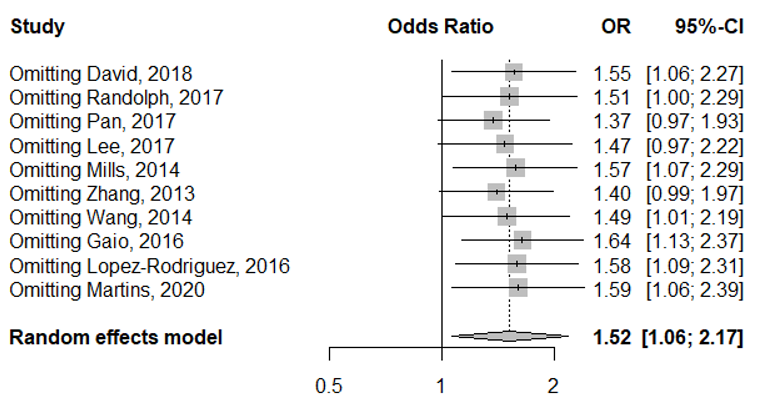 |
| Dominant model (CC/CT *vs* TT) |
| 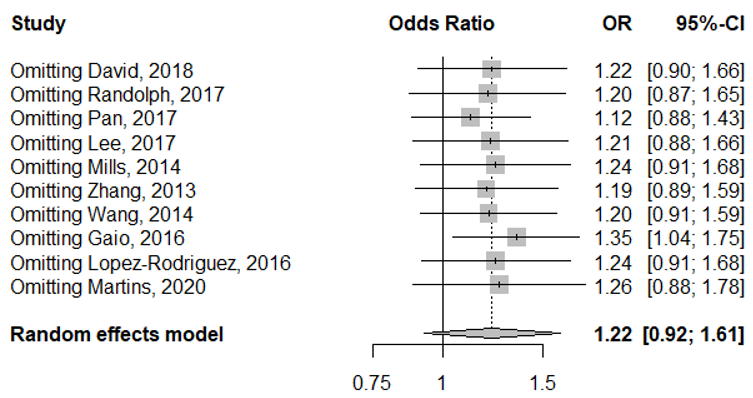 |
| Homozygous model (CC *vs* TT) |
| 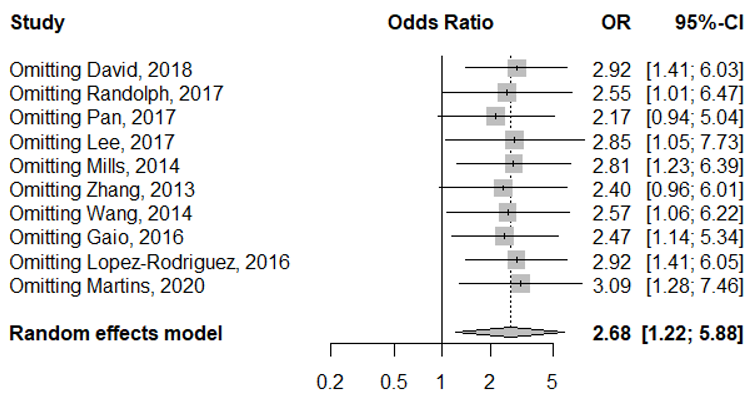 |
| Heterozygous model (CT *vs* TT) |
| 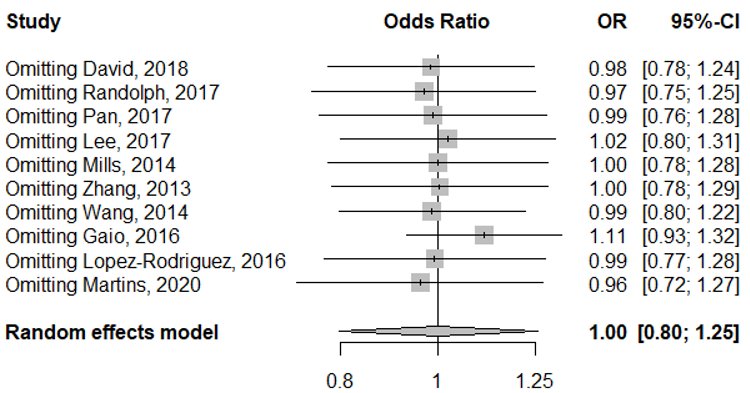 |

1. Funnel plots

| Allelic model (C *vs* T) |
| --- |
| 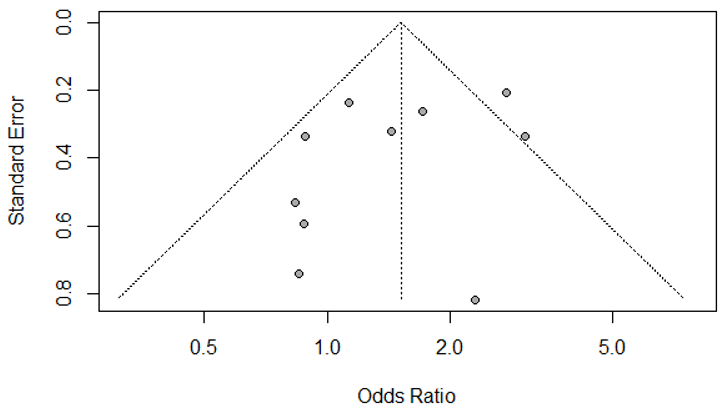 |
| Dominant model (CC/CT *vs* TT) |
| 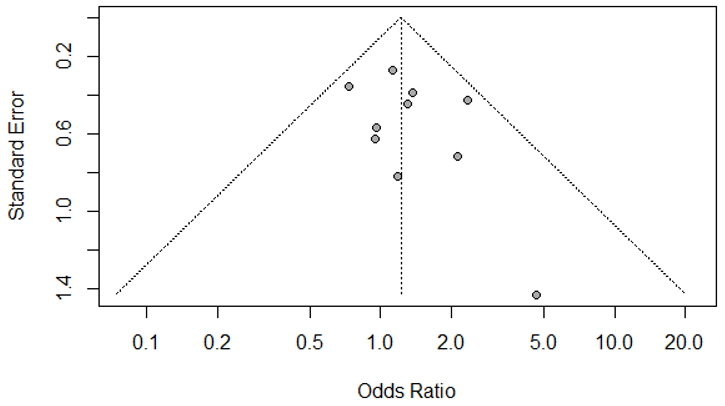 |
| Homozygous model (CC *vs* TT) |
| 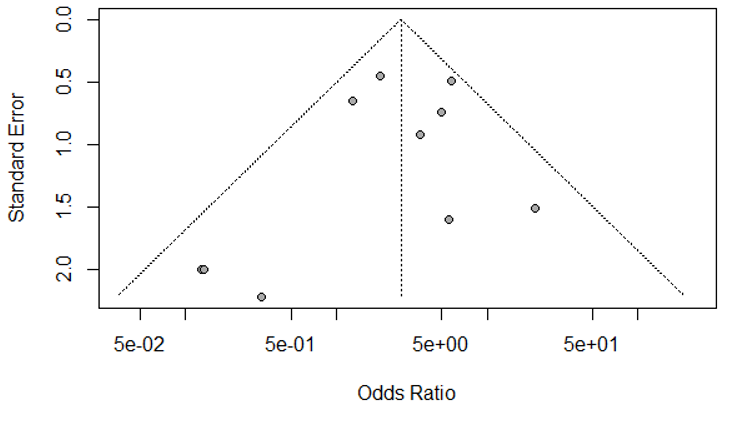 |
| Heterozygous model (CT *vs* TT) |
| 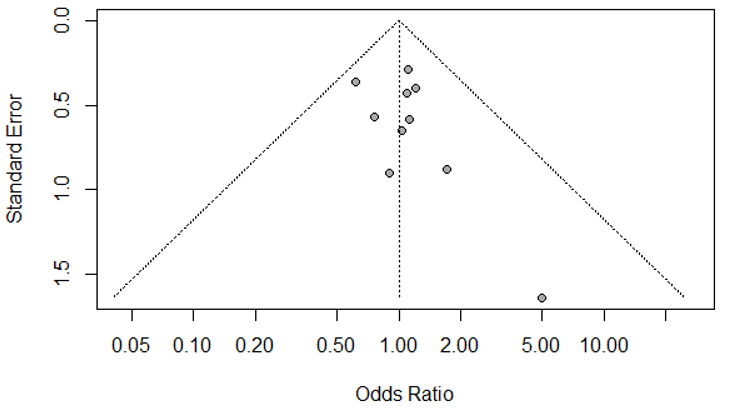 |

**Results meta-analysis CCR5 – rs333**

1. Study characteristics

| **Study** | **Ethnicity** | **Assessment of severity** | **Cases (n)** | **Controls (n)** | **rs333 cases** | | | | **rs333 controls** | | | | $\boldsymbol{P}_{\boldsymbol{HWE}}\boldsymbol{*}$ |
| --- | --- | --- | --- | --- | --- | --- | --- | --- | --- | --- | --- | --- | --- |
|  |  |  |  |  | **-/-** | **-/+** | **+/+** | **MAF** | **-/-** | **-/+** | **+/+** | **MAF** |  |
| Matos et al., 2019 | Hispanic | Severity indicators among H and non-H patients | 279 | 153 | 0 | 29 | 250 | 0.05 (-) | 0 | 12 | 141 | 0.04 (-) | >.99 |
| Matos et al., 2019 | Hispanic | Mortality among H and non-H patients | 106 | 326 | 0 | 8 | 98 | 0.04 (-) | 0 | 33 | 293 | 0.05 (-) | >.99 |
| Falcon et al., 2015 | Caucasian | Mortality among H and non-H patients | 11 | 160 | 1 | 3 | 7 | 0.23 (-) | 2 | 17 | 141 | 0.07 (-) | 0.13 |
| Maestri et al., 2015 | Admixed | Hospital admission among H and non-H patients | 156 | 174 | 0 | 8 | 148 | 0.03 (-) | 1 | 13 | 160 | 0.04 (-) | 0.27 |
| Sironi et al., 2014 | Caucasian | Hospital admission among H and non-H patients | 21 | 8 | 0 | 0 | 21 | 0.00 (-) | 0 | 1 | 7 | 0.06 (-) | >.99 |

*HWE calculated within controls.

H: hospitalized; HWE: Hardy-Weinberg Equilibrium; MAF: minor allele frequency.

1. Meta-analysis of the main outcome per study (main analysis)

| Allelic model (- *vs* +) |
| --- |
| 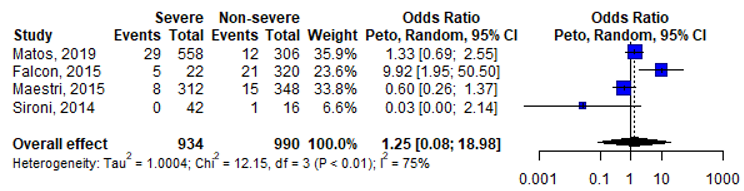 |
| Dominant model (--/-+ *vs* ++) |
| 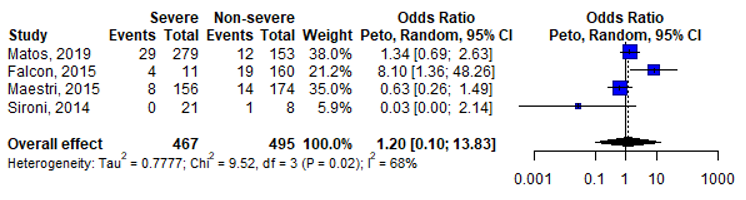 |
| Homozygous model (-- *vs* ++) |
| NA |
| Heterozygous model (-+ *vs* ++) |
| 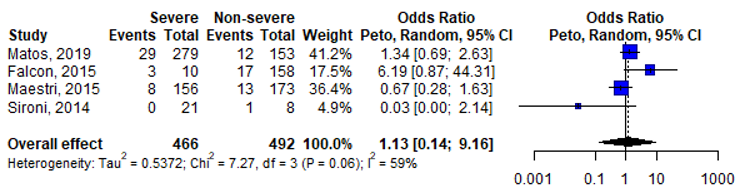 |

1. Leave-one-out analysis (sensitivity analysis)

| Allelic model (- *vs* +) |
| --- |
| 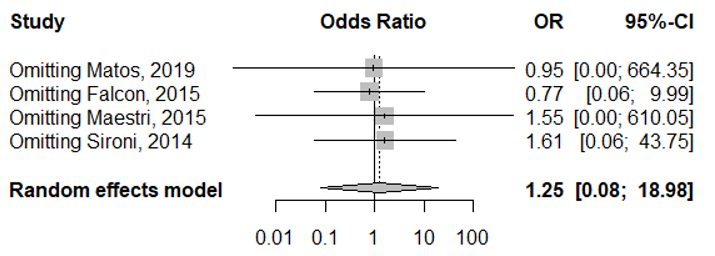 |
| Dominant model (--/-+ *vs* ++) |
| 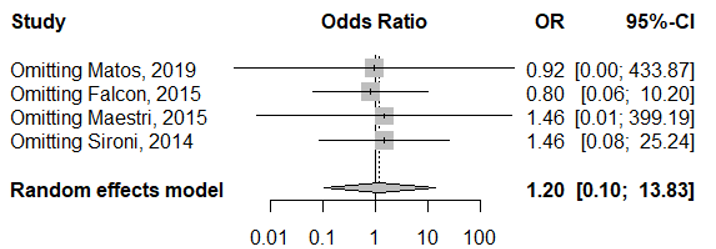 |
| Homozygous model (-- *vs* ++) |
| NA |
| Heterozygous model (-+ *vs* ++) |
| 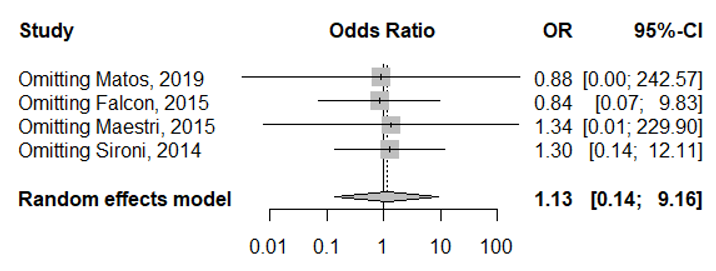 |

1. Funnel plots

| Allelic model (- *vs* +) |
| --- |
| 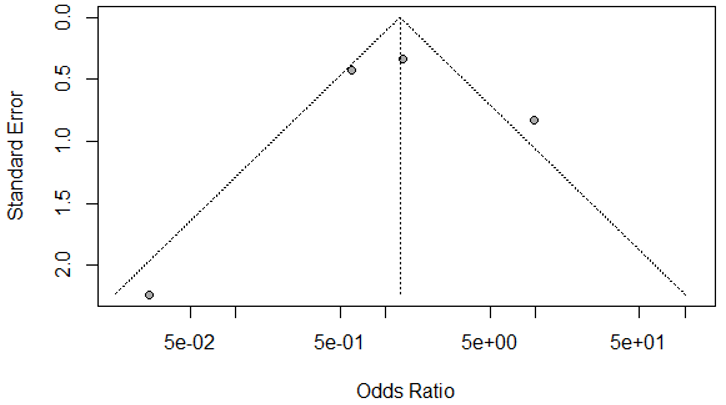 |
| Dominant model (--/-+ *vs* ++) |
| 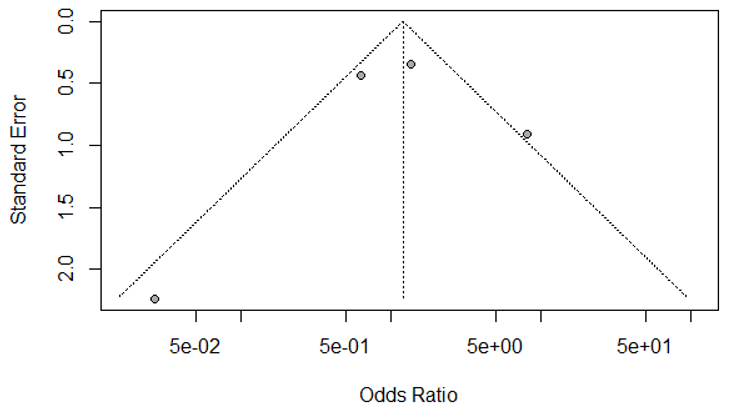 |
| Homozygous model (-- *vs* ++) |
| NA |
| Heterozygous model (-+ *vs* ++) |
| 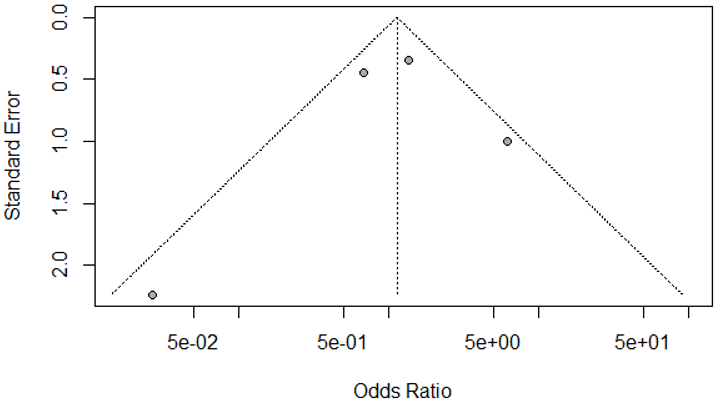 |

**Results meta-analysis FCGR2A – rs1801274**

1. Study characteristics

| **Study** | **Ethnicity** | **Assessment of severity** | **Cases (n)** | **Controls (n)** | **rs1801274 cases** | | | | **rs1801274 controls** | | | | $\boldsymbol{P}_{\boldsymbol{HWE}}\boldsymbol{*}$ |
| --- | --- | --- | --- | --- | --- | --- | --- | --- | --- | --- | --- | --- | --- |
|  |  |  |  |  | **AA** | **AG** | **GG** | **MAF** | **AA** | **AG** | **GG** | **MAF** |  |
| Chatzopoulou et al., 2019 | Caucasian | Severity indicators (e.g. ICU, ventilation, mortality) among H patients | 59 | 51 | 19 | 28 | 12 | 0.44 (G) | 19 | 24 | 8 | 0.39 (G) | >.99 |
| Chatzopoulou et al., 2019 | Caucasian | Mortality among H patients | 19 | 91 | 4 | 10 | 5 | 0.53 (G) | 34 | 42 | 15 | 0.40 (G) | 0.83 |
| Maestri et al., 2016 | Admixed | Hospital admission among H and non- H patients | 244 | 192 | 84 | 115 | 45 | 0.42 (G) | 73 | 80 | 39 | 0.41 (G) | 0.05 |
| Maestri et al., 2016 | Admixed | Mortality among H patients | 100 | 144 | 37 | 41 | 22 | 0.42 (G) | 45 | 74 | 23 | 0.43 (G) | 0.61 |
| Chan et al., 2011 | Asian | Severity indicators (e.g. ICU, ventilation, mortality) among H patients | 37 | 36 | 23 | 9 | 5 | 0.26 (G) | 16 | 17 | 3 | 0.32 (G) | >.99 |

*HWE calculated within controls.

H: hospitalized; HWE: Hardy-Weinberg Equilibrium; MAF: minor allele frequency.

1. Meta-analysis of the main outcome per study (main analysis)

| Main outcome per study – allelic model (G *vs* A) |
| --- |
| 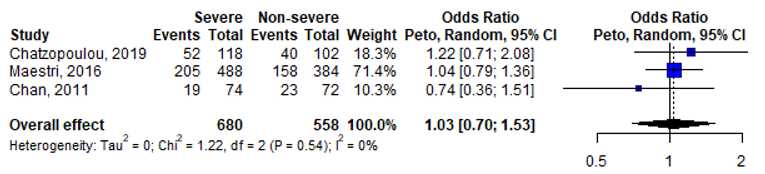 |
| Main outcome per study – dominant model (GG/AG *vs* AA) |
| 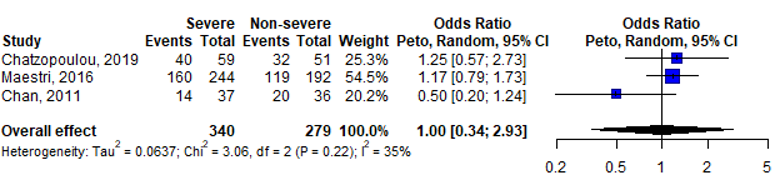 |
| Main outcome per study – homozygous model (GG *vs* AA) |
| 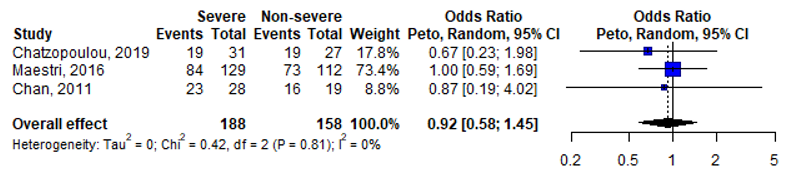 |
| Main outcome per study – heterozygous model (AG *vs* AA) |
| 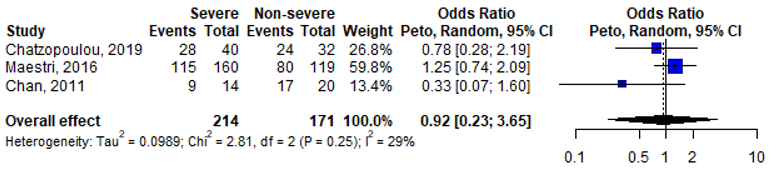 |

1. Leave-one-out analysis (sensitivity analysis)

| Allelic model (G *vs* A) |
| --- |
| 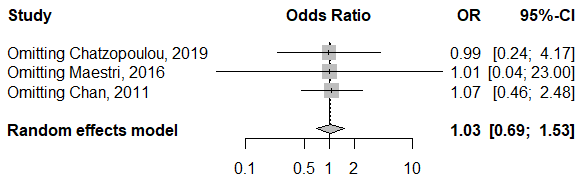 |
| Dominant model (GG/AG *vs* AA) |
| 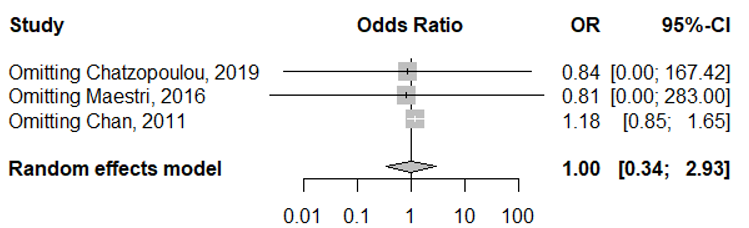 |
| Homozygous model (GG *vs* AA) |
| 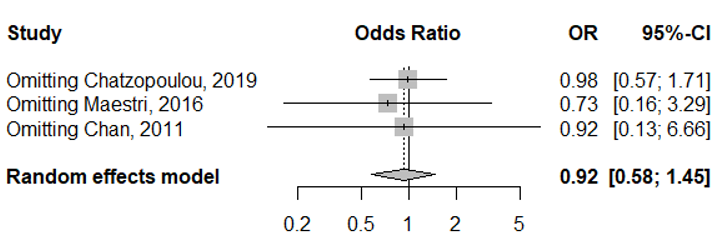 |
| Heterozygous model (AG *vs* AA) |
| 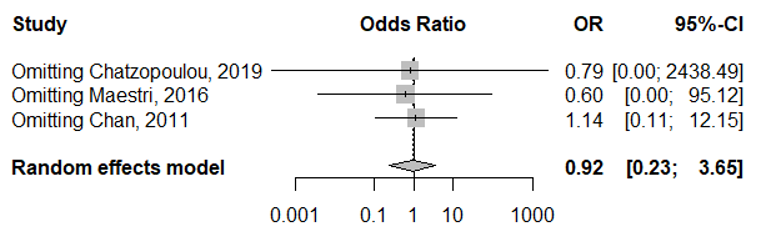 |

1. Funnel plots

| Allelic model (G *vs* A) |
| --- |
| 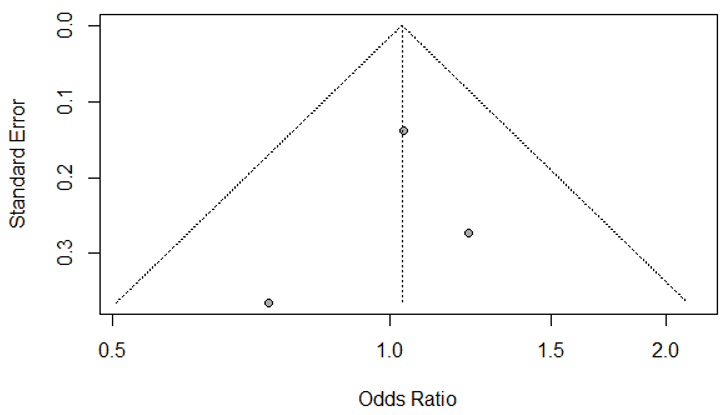 |
| Dominant model (GG/AG *vs* AA) |
| 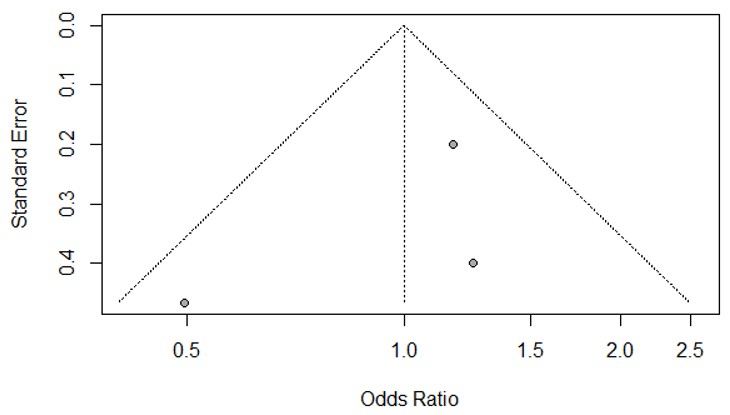 |
| Homozygous model (GG *vs* AA) |
| 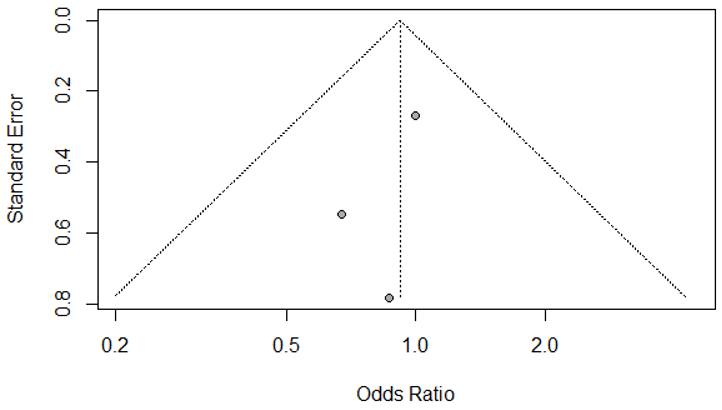 |
| Heterozygous model (AG *vs* AA) |
| 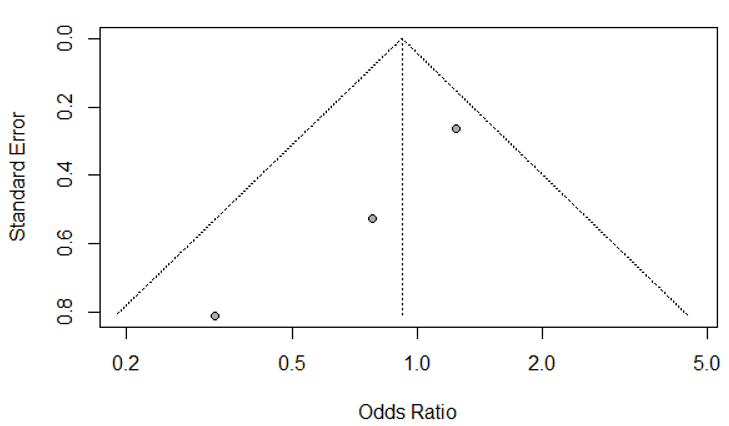 |

**Results meta-analysis IFITM3 – rs34481144**

1. Study characteristics

| **Study** | **Ethnicity** | **Assessment of severity** | **N cases** | **N controls** | **rs34481144 cases** | | | | **rs34481144 controls** | | | | $\boldsymbol{P}_{\boldsymbol{HWE}}\boldsymbol{*}$ |
| --- | --- | --- | --- | --- | --- | --- | --- | --- | --- | --- | --- | --- | --- |
|  |  |  |  |  | **AA** | **AG** | **GG** | **MAF** | **AA** | **AG** | **GG** | **MAF** |  |
| David et al., 2018 | Caucasian | Severity indicators among H and non-H patients | 22 | 19 | 2 | 6 | 14 | 0.23 (A) | 2 | 11 | 6 | 0.39 (A) | 0.63 |
| Allen et al., 2017 | Admixed | Mortality among H patients | 17 | 248 | 3 | 11 | 3 | 0.50 (A) | 35 | 95 | 118 | 0.33 (A) | 0.03 |
| Allen et al., 2017 | Admixed | Severity indicators among H and non-H patients | 9 | 77 | 3 | 3 | 3 | 0.50 (A) | 1 | 24 | 52 | 0.17 (A) | 0.68 |
| Martins et al., 2020 | Admixed | Severity indicators among H and non-H patients | 222 | 92 | 20 | 95 | 107 | 0.30 (A) | 11 | 38 | 43 | 0.33 (A) | 0.64 |
| Martins et al., 2020 | Admixed | Mortality among H and non-H patients | 82 | 232 | 7 | 37 | 38 | 0.31 (A) | 24 | 96 | 112 | 0.31 (A) | 0.65 |

*HWE calculated within controls.

H: hospitalized; HWE: Hardy-Weinberg Equilibrium; MAF: minor allele frequency.

1. Meta-analysis of the main outcome per study (main analysis)

| Main outcome per study – allelic model (A *vs* G) |
| --- |
| 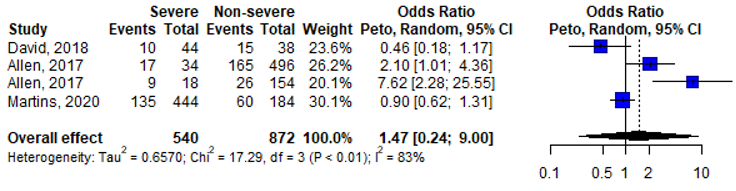 |
| Main outcome per study – dominant model (AA/AG *vs* GG) |
| 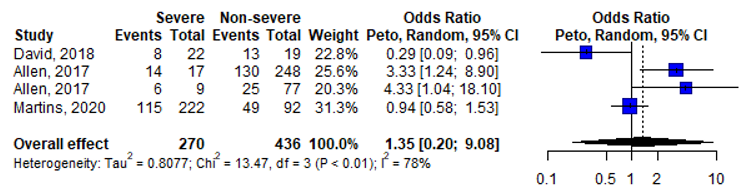 |
| Main outcome per study – homozygous model (AA *vs* GG) |
| NA |
| Main outcome per study – heterozygous model (AG *vs* GG) |
| 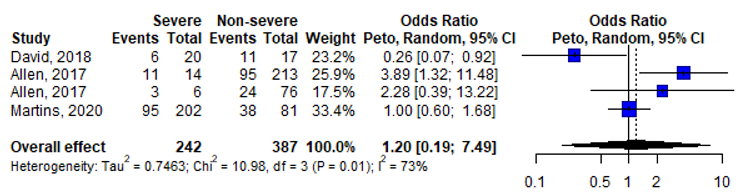 |

1. Leave-one-out analysis (sensitivity analysis)

| Allelic model (A *vs* G) |
| --- |
| 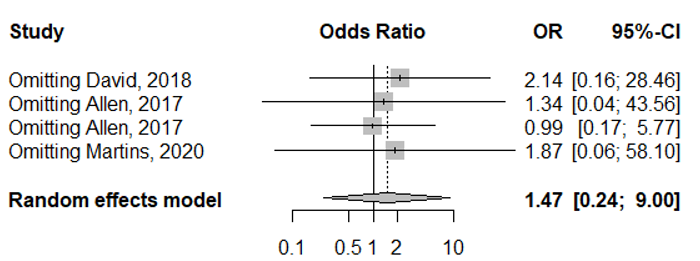 |
| Dominant model (AA/AG *vs* GG) |
| 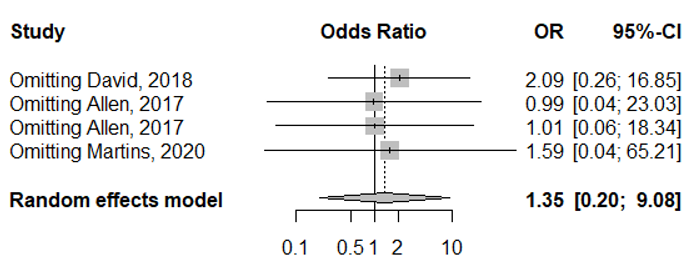 |
| Homozygous model (AA *vs* GG) |
| NA |
| Heterozygous model (AG *vs* GG) |
| 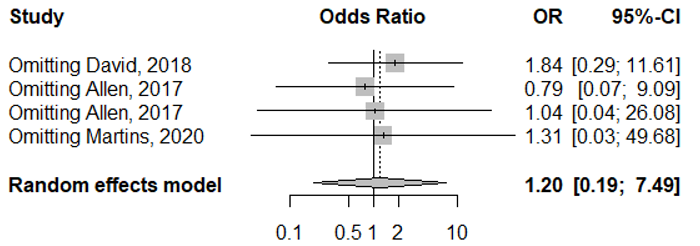 |

1. Funnel plots

| Allelic model (A *vs* G) |
| --- |
| 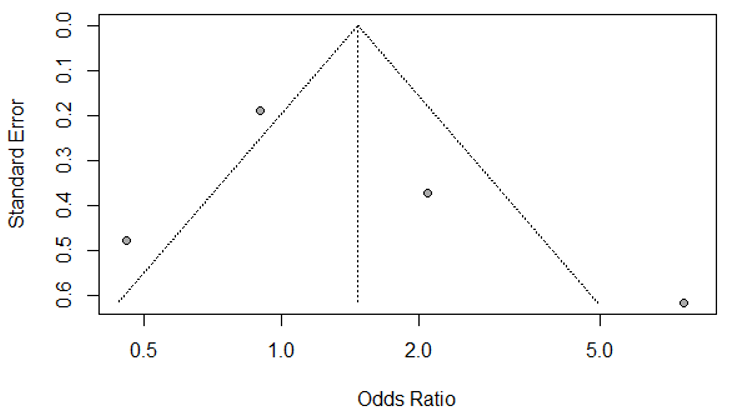 |
| Dominant model (AA/AG *vs* GG) |
| 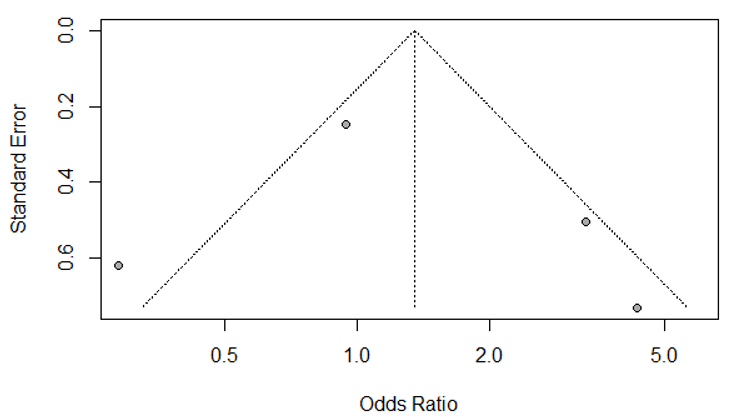 |
| Homozygous model (AA *vs* GG) |
| NA |
| Heterozygous model (AG *vs* GG) |
| 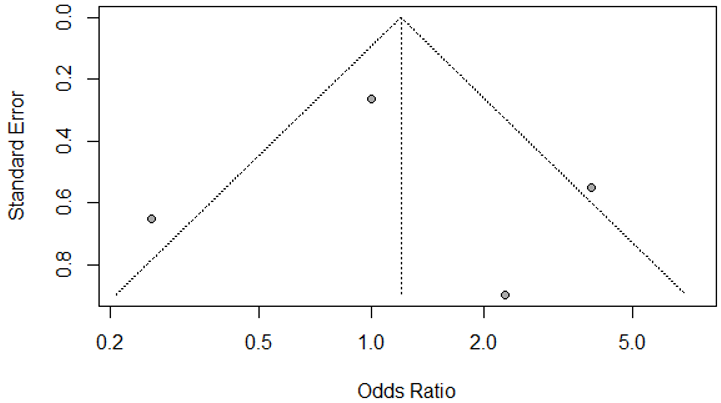 |
