# Supplementary material for: A systematic review and meta-analysis of host genetic factors associated with influenza severity
Source: BMC Genomics. 2021 Dec 20;22:912. doi: 10.1186/s12864-021-08240-7 (PMC8686082; doi:10.1186/s12864-021-08240-7)
Supplement: Supplementary file 3 — Additional file 3. Search strategy using the MEDLINE and EMBASE databases, eligibility criteria, and risk of bias score to assess the quality of included studies for the systematic review and meta-analysis of host genetic factors and influenza disease severity. [file 12864_2021_8240_MOESM3_ESM.docx]

**Additional file 3**

**A meta-analysis of host genetic factors associated with influenza severity**

Nina Van Goethem^1,2, *^, Célestin Danwang^2^, Nathalie Bossuyt^1^, Herman Van Oyen^1,3^, Nancy H. C. Roosens^4,£^, Annie Robert^2,£^

^1^Scientific Directorate of Epidemiology and public health, Sciensano, J. Wytsmanstraat 14, 1050 Brussels, Belgium.

^2^Department of Epidemiology and Biostatistics, Institut de recherche expérimentale et clinique, Faculty of Public Health, Université catholique de Louvain, Clos Chapelle-aux-champs 30, 1200 Brussels, Belgium.

^3^Department of Public Health and Primary Care, Ghent University, De Pintelaan 185, 9000 Ghent, Belgium.

^4^Transversal activities in Applied Genomics, Sciensano, J. Wytsmanstraat 14, 1050 Brussels, Belgium.

^£^Shared last author

**Search strategy for the systematic review and meta-analysis of host genetic factors and influenza severity using the MEDLINE and EMBASE databases**

1. MEDLINE

| **Domain** | **Search terms** |
| --- | --- |
| Host genetics | ("Human Genetics"[Mesh] OR "host genetics"[TIAB] OR "host susceptibility"[TIAB] OR "host genes"[TIAB] OR "Polymorphism, Genetic"[Mesh] OR "polymorphism"[TIAB] OR "Genetic Variation"[Mesh] OR "single-nucleotide polymorphism"[TIAB] OR "mutation"[TIAB] OR "Genetic Predisposition to Disease"[Mesh]) |
| Human influenza | ("Influenza, Human"[Mesh] OR "influenza"[TIAB]) |
| Severe infection | ("Virulence"[Mesh] OR "virulence"[TIAB] OR "sever*"[TIAB] OR "pathogenic*"[TIAB] OR "death"[TIAB] OR "fatal*"[TIAB] OR "complication*"[TIAB] OR "natural course"[TIAB] OR "Virulence"[Mesh] OR "Severity of Illness Index"[Mesh]) |

Search string:

("Human Genetics"[Mesh] OR "host genetics"[TIAB] OR "host susceptibility"[TIAB] OR "host genes"[TIAB] OR "Polymorphism, Genetic"[Mesh] OR "polymorphism"[TIAB] OR "Genetic Variation"[Mesh] OR "single-nucleotide polymorphism"[TIAB] OR "mutation"[TIAB] OR "Genetic Predisposition to Disease"[Mesh]) AND ("Influenza, Human"[Mesh] OR "influenza"[TIAB]) AND ("Virulence"[Mesh] OR "virulence"[TIAB] OR "sever*"[TIAB] OR "pathogenic*"[TIAB] OR "death"[TIAB] OR "fatal*"[TIAB] OR "complication*"[TIAB] OR "natural course"[TIAB] OR "Virulence"[Mesh] OR "Severity of Illness Index"[Mesh] )

*818 hits (Species=Humans) on 28/02/2020*

*96 hits (Species=Humans, from 2020/2/1 - 3000/12/12) on 05/07/2021*

1. EMBASE

Search string:

('influenza virus'/exp OR 'influenza') AND ('human genetics'/exp OR 'human genetics' OR 'host genetics' OR 'host susceptibility' OR 'host genes' OR 'gene mutation'/exp OR 'snp'/exp OR 'genetic predisposition'/exp) AND ('disease severity'/exp OR 'severe' OR 'severity' OR 'virulence'/exp OR 'virulence' OR 'infection complication'/exp OR 'complications' OR 'pathogenic' OR 'severity of illness index'/exp) AND 'human'/de

*975 hits on 28/02/2020*

('influenza virus'/exp OR 'influenza') AND ('human genetics'/exp OR 'human genetics' OR 'host genetics' OR 'host susceptibility' OR 'host genes' OR 'gene mutation'/exp OR 'snp' OR 'genetic predisposition'/exp) AND ('disease severity'/exp OR 'severe' OR 'severity' OR 'virulence'/exp OR 'virulence' OR 'infection complication'/exp OR 'complications' OR 'pathogenic' OR 'severity of illness index'/exp) AND 'human'/de AND [1-2-2020]/sd

*294 hits on 05/07/2021*

**Eligibility criteria for the systematic review and meta-analysis of host genetic factors and influenza severity**

Articles were included only if it was a primary study that:

- included cases who are individuals with a severe laboratory-confirmed influenza infection (end-point should be susceptibility to *severe* influenza infection as compared to mild/asymptomatic influenza infection);
- defined severity as 1) severity indicators (e.g., ICU admission, invasive ventilation, or mortality) among hospitalized influenza positive patients; 2) mortality among hospitalized influenza positive patients; 3) mortality among hospitalized and ambulant influenza positive patients; 4) severity indicators (e.g., ICU admission, invasive ventilation, or mortality) among hospitalized and ambulant influenza positive patients; or 5) hospital admission among ambulant influenza positive patients;
- assessed human genetic polymorphisms or genetic variants (only bi-allelic single nucleotide polymorphisms (SNPs) or bi-allelic insertion-deletion marker types have been considered).

Articles were excluded when it was a study that:

- had not been carried out on humans, such as animal studies, *in vitro* and *in silico* studies;
- did not include a study population with a laboratory-confirmed influenza infection;
- were categorized as case reports, letters, comments, reviews, or editorials;
- had no appropriate study design, i.e. not being considered as a case-control or cohort study;
- only assessed the susceptibility to the influenza infection itself as compared to influenza negative or healthy controls;
- only assessed other marker types, such as microsatellites, short tandem repeats, aggregated haplotypes, or gene expression profiles;
- had no English full text available.

**Risk of bias score to assess the quality of included studies**

The Confounding-Selection-Information (CSI) bias score developed by Patarcic *et al* [1] was used to evaluate the methodological quality of the included studies. The elements of the score were developed on the basis of several existing assessment scores, including Venice criteria for assessing cumulative epidemiologic evidence in genetic associations [2], Newcastle-Ottawa case-control scale [3], and the Cochrane risk of bias tool [4].

| Domain | Level A grade | Level B grade | Level C grade |
| --- | --- | --- | --- |
| Confounding risk | No apparent confounding (or possible confounding properly adjusted for) AND no indication of population stratification | Some degree of confounding possible/probable OR study performed in obviously admixed population | Detectable levels of confounding OR indication of strong stratification |
| Selection bias risk | Controls drawn from general population AND satisfying Hardy-Weinberg equilibrium (HWE) | Controls drawn from structured sampling frame (hospital, clinic, or health care programme-based) AND in HWE | No description on controls recruitment OR controls failed HWE |
| Information bias risk | I1: Cases: status verified by highly specific molecular methods (antigen test, PCR)  I2: Controls: status verified by highly specific molecular methods (antigen test, PCR)  I3: Genotyping: favourable quality control estimates given, subset or total dataset replicated | I1: Cases status established on the basis of guidelines, clinical status, or less specific methods (isolation, smears and microbiological cultures)  I2: Controls status inferred from medical records only (no history of disease)  I3: Partial genotyping quality control results | I1: No clear case definition provided  I2: No description of disease status in controls (“healthy” controls)  I3: No indication of genotyping reproducibility |

**References**

1. Patarčić I, Gelemanović A, Kirin M, Kolčić I, Theodoratou E, Baillie KJ, et al. The role of host genetic factors in respiratory tract infectious diseases: systematic review, meta-analyses and field synopsis. Sci Rep. 2015;5: 16119. doi:10.1038/srep16119

2. Ioannidis JPA, Boffetta P, Little J, O’Brien TR, Uitterlinden AG, Vineis P, et al. Assessment of cumulative evidence on genetic associations: interim guidelines. Int J Epidemiol. 2008;37: 120–132. doi:10.1093/ije/dym159

3. Wells G, Shea B, O’Connell D, Peterson J, Welch V, Losos M, et al. The Newcastle-Ottawa Scale (NOS) for assessing the quality of nonrandomised studies in meta-analyses. 2013. Available: http://www.ohri.ca/programs/clinical_epidemiology/oxford.asp

4. Higgins, J.P.T. Cochrane handbook for systematic reviews of interventions version 5.1.0. 2011. Available: http://www.cochrane-handbook.org
